# Infective endocarditis presenting initially with ileus complicated by dehiscence of annuloplasty ring

**DOI:** 10.1186/s12872-015-0113-2

**Published:** 2015-10-14

**Authors:** Takao Konishi, Hiroshi Nishihara, Tadashi Ito, Yoshiaki Tanaka

**Affiliations:** Department of Cardiology, Hokkaido Cardiovascular Hospital, 1–30, West 13, South 27, Chuou-ku, Sapporo, 064–8622 Japan; Department of Translational Pathology, Hokkaido University, Graduate School of Medicine, Sapporo, Japan; Department of Cardiovascular Surgery, Self Defence Forces Central Hospital, Tokyo, Japan; Department of Cardiovascular Surgery, Saitama Eastern Cardiovascular Hospital, Saitama, Japan

**Keywords:** Infective endocarditis, Annuloplasty ring dehiscence, Systemic embolism, Mesenteric embolism, Infective ileus

## Abstract

**Background:**

Infective endocarditis (IE) on an annuloplasty ring dehiscence is uncommon after mitral valve repair.

**Case Presentation:**

A 53-year-old man underwent mitral annuloplasty with a 24-mm ring for posterior mitral valve prolapse. He underwent repeat valve repair for recurrent mitral valve regurgitation 4 years later. He was re-hospitalised complaining of vomiting, nausea, general fatigue and left abdominal pain 2 months later, and presented with low-grade fever, leukocytosis and an elevated blood concentration of C-reactive protein. An abdominal computed tomography scan showed multiple embolisms in the liver, kidney and spleen. Transoesophageal echocardiography revealed mitral annuloplasty ring dehiscence and vegetations consistent with IE. The infected annuloplasty ring and vegetations were surgically excised. Blood cultures grew coagulasenegative staphylococcus aureus, consistent with the excised mitral valve histology. The postoperative course was uneventful, without recurrence of IE.

**Conclusions:**

Embolic ileus as initial manifestation of IE is rare and might confuse the diagnosis and delay its management. Gastrointestinal signs and symptoms may be the initial manifestations of systemic embolization from infective endocarditis. Transoesophageal echocardiography effectively identified the presence of vegetations and mitral annuloplasty ring dehiscence.

## Background

Systemic embolization is a major complication observed in up to 55 % of patients presenting with infective endocarditis (IE), and large vegetations are associated with a high incidence of fatal or non-fatal embolic events [1, 2]. An early diagnosis followed by surgical treatment is of critical importance to reduce the rate of complications. In contrast to the brain, involved in nearly 50 % of embolic events originating from left or right-sided vegetations, [3] systemic embolisms caused by IE rarely present initially with symptomatic abdominal manifestations. We present a case of IE in a middle-aged man first presenting with an ileus, subsequently complicated by the dehiscence of an annuloplasty ring.

## Case presentation

A 53-year-old man presented to the emergency department complaining of pain in the left iliac fossa of 6 h-duration, along with fever, nausea, anorexia and general fatigue, though without cardiovascular manifestations. He had undergone mitral annuloplasty with a 24-mm ring for posterior mitral valve prolapse 4 years earlier, followed by further mitral valve repair for recurrent mitral regurgitation (MR). The histopathological examination had then revealed no IE, but severe myxomatous degeneration of a mitral tendon, thought to have caused the rupture of a mitral chordae and precipitated MR. After surgical repair, the patient chose to leave the hospital prematurely despite the persistence of a low-grade fever.

**Fig. 1:**
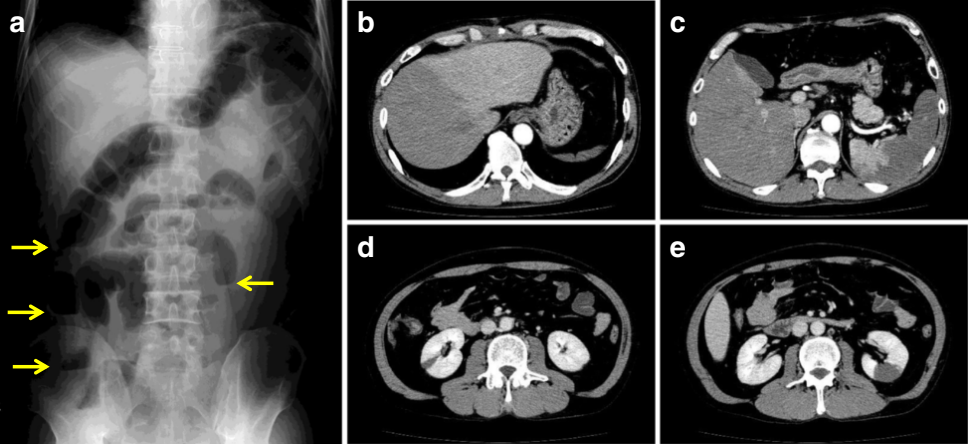
**a**. Dilated intestines and niveau (arrows), consistent with ileus, are present on this plain radiograph of the abdomen. **b**–**e**. 64-row, multi-detector, abdominal computed tomography showing hepatic **b**, splenic **c** and bilateral renal **d** & **e** infarctions

**Fig. 2:**
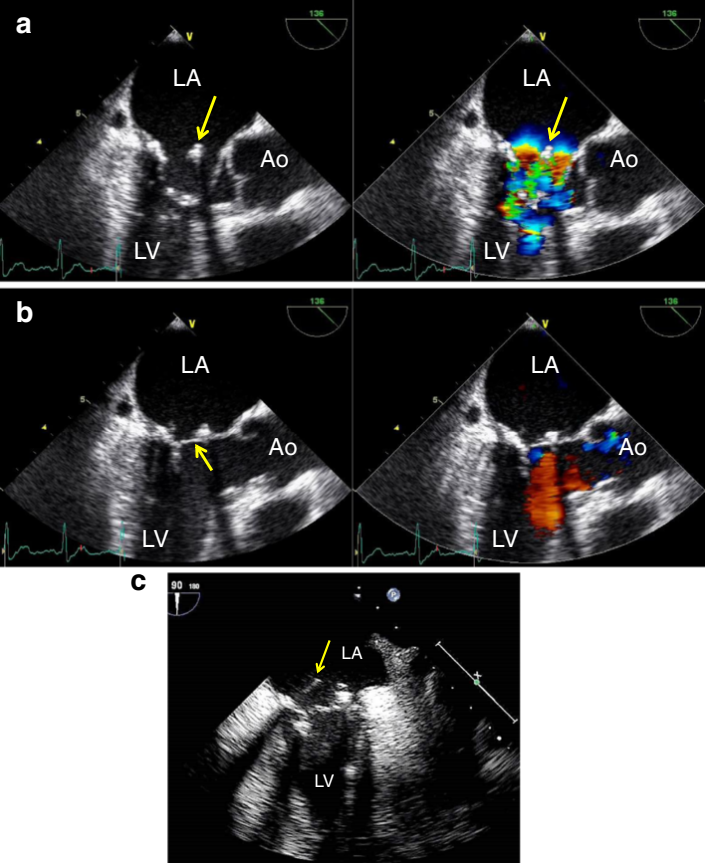
Transoesophageal echocardiography. **a**. The mitral annuloplasty ring was detached from the mitral annulus at a 136° angle in early diastole (*arrow*). LA = left atrium; LV = left ventricle; Ao = Aorta. **b**. The mitral valve is re-attached to the annuloplasty ring in early systole (*arrow*); trivial MR is present on colour Doppler echocardiography. **c**. Transoesophageal, two-chamber echocardiogram showing 15-mm long, highly mobile vegetations (*arrow*)

When he returned, 2 months later, his examination revealed a clear mental status, a body temperature a 37.6 °C, a blood pressure at 87/55 mmHg, and a heart rate at 85 bpm. The percutaneous oxygen saturation on room air was 95 %. He had no audible pathologic heart murmur, no manifestation of cardiac decompensation and no sign of thromboembolism. The abdomen, however, was slightly distended, with a painful left iliac fossa and rebound tenderness on palpation. The white blood cell count was 25.2 × 10^3^/mm^3^ (granulocytes 88.9 %, lymphocytes 3.5 %) and 235 × 10^3^/mm^3^ platelets, the blood haemoglobin concentration was 11.1 g/dl, and C-reactive protein concentration was 6.36 mg/dl. A plain film of the abdomen showed dilated small and large bowels with niveau formation (Fig. 1a). A tentative diagnosis of ileus was made initially by consultant gastroenterologists. An abdominal computed tomography with contrast showed low-density areas within the liver, spleen and both kidneys (Fig. 1b–e), prompting the patient’s referral to a cardiologist for evaluation of multiple embolisms, putatively caused by IE. Transthoracic echocardiography detected no significant MR, though mitral valve thickening was present, which was not observed after the previous mitral valve repair. The left ventricular end-diastolic and end-systolic diameters were 47 and 31 mm, respectively and ejection fraction was 63 %. A transoesophageal echocardiogram was performed, which revealed a prominently mobile vegetation and dehiscence of the annuloplasty ring from the anterior mitral annulus (Fig. 2). Despite a separation of the ring from the anterior aspect of the mitral annulus during diastole, no significant MR was observed. The anterior dehiscent part of the annuloplasty ring was located in the middle of the mitral orifice and consecutively in touch with the anterior leaflet, not the anterior annulus, during systole, when the leaflets were pushed towards the atrium. Accordingly, during diastole, the leaflet opened towards the ventricle and the annuloplasty ring remained in its position, detached from the leaflet. Active IE was diagnosed as the cause of multiple embolisms and septicaemia. Intravenous gentamicin, 40 mg t.i.d, and ampicillin, 1.5 g b.i.d, were administered immediately after the admission. Brain computed tomography and magnetic resonance imaging showed neither cerebral infarction nor aneurysm.

**Fig. 3:**
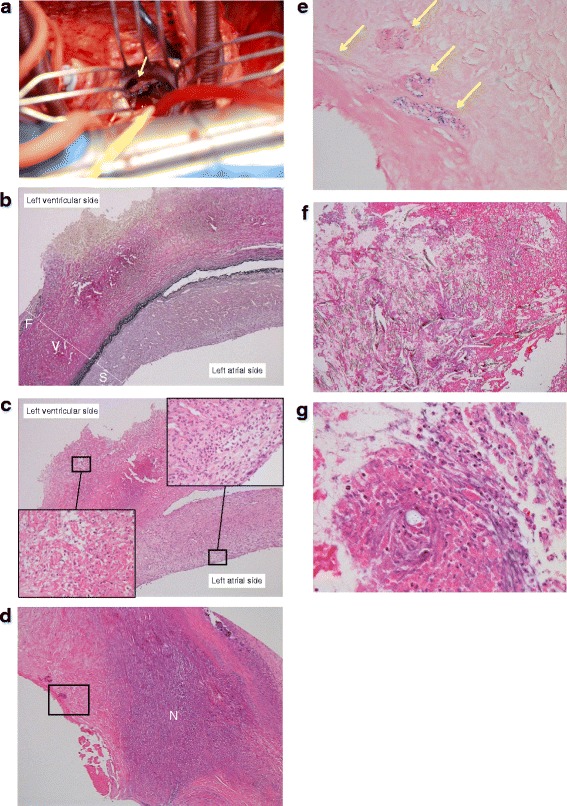
Intraoperative inspection and histopathology of the mitral valve. **a**. Intraoperative appearance of anterior mitral annuloplasty ring dehiscence. **b**. Low-power (X 50 original magnification) microphotograph of tissue destruction in the 3 layers of the anterior mitral valve observed after *elastica* van Gieson staining. F = *lamina fibrosa*; V = *lamina ventricularis*; S = *lamina spongiosa*. **c**. High-power (X400 original magnification) microphotograph of neutrophils infiltration on left ventricular and atrial aspects of mitral valve observed after haematoxylin and eosin staining. Similar observations (not shown) were made on the posterior mitral valve. **d**. Widely necrotic (N) anterior mitral valve. **e**. High-power (X400 original magnification) microphotograph in the area surrounded by black line in Fig. 3d showing the accumulation of gram-positive *staphylococci* (*arrows*). **f**. Low-power (X50 original magnification) microphotograph of extensive inflammation and necrosis around the suture threads in the anterior mitral valve observed after haematoxylin and eosin staining. **g**. High-power (X400 original magnification) microphotograph showing necrosis and the infiltration of neutrophils

After the patient’s admission to the cardiovascular surgery department, his body temperature rose to 39.4 °C. He underwent mitral valve replacement 2 days later for management of annuloplasty ring dehiscence, impending recurrent embolization and uncontrolled infection. Intraoperative inspection confirmed the presence of a dehiscent anterior portion of the mitral annuloplasty ring (Fig. 3a). High- and low-power histopathologic microphotographs showed the destruction of the three layers of the anterior mitral leaflet (Fig. 3b–e), with Gram positive *cocci* along with severe inflammation and necrosis of the valvular tissue (Fig. 3d). All the blood cultures were positive for coagulase-negative *staphylococcus capitis*, consistent with the histopathology. Four days after the surgery, intravenous ampicillin was replaced with vancomycin, 0.5 g t.i.d because of resistance to ampicillin.

The postoperative course was uneventful with resolution of the manifestations of abdominal disease without further treatment and, over a follow-up of 18 months, the patient remained free from recurrence of IE.

## Discussion

This case illustrates atypical characteristics of IE. First, systemic embolization, in this patient, presented initially as an ileus. Second, depending on the degree of detachment of the annuloplasty ring from the mitral annulus, the amount of MR was insufficient to raise the suspicion of a dehiscence. To the best of our knowledge, this postoperative complication of repeat mitral valve repair has not been previously described in the English language medical literature.

The diagnosis of IE is often challenging, as its symptomatology is variable. Its incidence requiring surgical intervention after mitral valve repair is <1 % [4]. Embolic events occur in 25 % of patients presenting with mitral valve endocarditis [5]. Symptomatic systemic embolisms more often involve the central nervous system than other organs, such as the spleen, kidneys, lung or liver [3]. Consequently, predominant or initial abdominal symptoms in absence of neurological manifestations may confuse the diagnosis and delay the treatment of IE.

In this patient, the ileus was probably not due to the IE and direct embolization but a reflectory paralysis of the intestine after embolization to liver, spleen and kidneys as described in some reports [6–8]. Actually, computed tomography in this case could not detect obvious embolization to any mesenteric arteries though we could not deny micro-embolization.

This patient presented an unusual morphologic type of annuloplasty ring dehiscence on transoesophageal echocardiogram (Fig. 2). The dehiscence is usually the cause of significant systolic MR, due to the gap between the ring and the mitral annulus [9]. In this case, the dehiscence was not fully appreciated initially because of the absence of prominent MR and because of a technically poor transthoracic echocardiogram. Since more detailed information can be obtained from a transoesophageal study, patients with suspected IE after mitral valve repair should undergo the procedure in search of ring dehiscence or vegetations.

The poor infection control was probably the cause of this case’s IE and embolization. Despite the surgeon’s recommendation for investigation, the patient chose to leave the hospital even though he had a low grade fever and an inflammation in the blood exam during the previous admission for mitral annuloplasty. After discharge, the infection might cause a persistent inflammation of mitral valves, leading to the laxation of sutured portion and, eventually, to the ring dehiscence. High- and low-power histopathologic microphotographs showed the neutrophil infiltration and extensive necrosis around the suture threads in the anterior mitral valve (Fig. 3f & g). Although the ring size of 24 mm was thought to be appropriate during the first operation, it might be smaller than the optimal size, considering the subsequent clinical course.

Although the dehiscence of a mitral annuloplasty ring usually occurs posteriorly or laterally, [9] in this patient it occurred anteriorly, which may be explained by his clinical characteristics. First, the inflammation and destruction of the mitral valve tissue was histopathologically more prominent in the anterior than in the posterior annulus. Second, this patient presented with small amounts of mitral annular calcification, which is usually more prominent in the posterior annular region, and the cause of dehiscence of the sutures.

Coagulase-negative *staphylococci* are the main infectious organisms recovered from prosthetic valves, and uncommonly from native valves, although several reports have described their emerging importance as a cause of native valve IE [10]. The majority of patients presenting with native valve IE due to coagulase-negative *staphylococci* present with valvular abnormalities, mitral valve prolapse in particular [1]. Furthermore, the increase in invasive procedures is likely to increase the number of patients at risk of native valve IE due to coagulase-negative *staphylococci* [11]. The adhesion of *S. capitis* to foreign body surfaces is weak [12]. In addition, 24 % of patients with IE due to *S. captis* are resistant to methicillin [13]. Therefore, vancomycin or linezolid should be administered as early as possible when blood cultures grow *S. capitis* in a patient with native valve IE.

## Conclusions

Embolic ileus as initial manifestation of IE is rare and might confuse the diagnosis and delay its management. Gastrointestinal signs and symptoms may be the initial manifestations of systemic embolization from infective endocarditis. Transoesophageal echocardiography effectively identified the presence of vegetations and mitral annuloplasty ring dehiscence.

## Consent

Written informed consent was obtained from the patient for publication of this case report and any accompanying images.

## Abbreviations

IE: Infective endocarditis
MR: Mitral regurgitation
